# Analysis of mRNA m^6^A modification and mRNA expression profiles in middle ear cholesteatoma

**DOI:** 10.3389/fgene.2023.1188048

**Published:** 2023-08-07

**Authors:** Shumin Xie, Li Jin, Jun He, Jinfeng Fu, Tuanfang Yin, Jihao Ren, Wei Liu

**Affiliations:** ^1^ Hunan Provincial Key Lab, Department of Otolaryngology-Head and Neck Surgery, The Xiangya Hospital, Otolaryngology Institute of Major Diseases, Central South University, Changsha, Hunan, China; ^2^ Department of Otolaryngology-Head and Neck Surgery, The Second Xiangya Hospital, Central South University, Changsha, Hunan, China

**Keywords:** mRNA, m^6^A, methylation profile, expression profile, middle ear cholesteatoma

## Abstract

**Introduction:** Middle ear cholesteatoma is characterized by the hyperproliferation of keratinocytes. In recent decades, N^6^-methyladenosine (m^6^A) modification has been shown to play an essential role in the pathogenesis of many proliferative diseases. However, neither the m^6^A modification profile nor its potential role in the pathogenesis of middle ear cholesteatoma has currently been investigated. Therefore, this study aimed to explore m^6^A modification patterns in middle ear cholesteatoma.

**Materials and methods:** An m^6^A mRNA epitranscriptomic microarray analysis was performed to analyze m^6^A modification patterns in middle ear cholesteatoma tissue (*n* = 5) and normal post-auricular skin samples (*n* = 5). Gene Ontology (GO) and Kyoto Encyclopedia of Genes and Genomes (KEGG) pathway analyses were performed to predict the potential biological functions and signaling pathways underlying the pathogenesis of middle ear cholesteatoma. Subsequently, m^6^A modification levels were verified by methylated RNA immunoprecipitation–qPCR (MeRIP–qPCR) in middle ear cholesteatoma tissue and normal skin samples, respectively.

**Results:** A total of 6,865 distinctive m^6^A-modified mRNAs were identified, including 4,620 hypermethylated and 2,245 hypomethylated mRNAs, as well as 9,162 differentially expressed mRNAs, including 4,891 upregulated and 4,271 downregulated mRNAs, in the middle ear cholesteatoma group relative to the normal skin group. An association analysis between methylation and gene expression demonstrated that expression of 1,926 hypermethylated mRNAs was upregulated, while expression of 2,187 hypomethylated mRNAs and 38 hypermethylated mRNAs was downregulated. Moreover, GO analysis suggested that differentially methylated mRNAs might influence cellular processes and biological behaviors, such as cell differentiation, biosynthetic processes, regulation of molecular functions, and keratinization. KEGG pathway analysis demonstrated that the hypermethylated transcripts were involved in 26 pathways, including the Hippo signaling pathway, the p53 signaling pathway, and the inflammatory mediator regulation of transient receptor potential (TRP) channels, while the hypomethylated transcripts were involved in 13 pathways, including bacterial invasion of epithelial cells, steroid biosynthesis, and the Hippo signaling pathway.

**Conclusion:** Our study presents m^6^A modification patterns in middle ear cholesteatoma, which may exert regulatory roles in middle ear cholesteatoma. The present study provides directions for mRNA m^6^A modification-based research on the epigenetic etiology and pathogenesis of middle ear cholesteatoma.

## 1 Introduction

Acquired middle ear cholesteatoma is a benign collection of keratinized squamous epithelial cells. It can progressively develop and erode nearby bony structures, leading to facial paralysis, hearing loss, vestibular dysfunction, and various intracranial complications (Castle, 2018). The incidence of acquired middle ear cholesteatoma has been reported to be 9.2 per 100,000 people in Europe, 6 per 100,000 people in America, and even higher in Asia (Aquino et al., 2011; Kennedy and Singh, 2022). Four major theories, namely, squamous metaplasia, epithelial migration, basal hyperplasia, and retraction pocket theories, have been proposed for decades. However, the exact pathogenesis of acquired cholesteatoma is not fully understood.

RNA modifications play a pivotal role in regulating gene expression by adjusting RNA structure and function, and their dysregulation has been associated with a wide range of developmental and physiological abnormalities as well as various diseases (Tong et al., 2018). Currently, more than 100 chemical modifications have been identified in RNA (Frye et al., 2018). N^6^-methyladenosine (m^6^A) modification is the most prevalent and well-studied post-transcriptional RNA modification that affects multiple aspects of RNA metabolism, including splicing, processing, transport, transcription, and RNA stability (Liu and Pan, 2015; Meyer et al., 2015; Xiao et al., 2016; Zaccara et al., 2019; Zhou et al., 2015). m^6^A RNA modification is dynamically and reversibly regulated by the methyltransferase complex, demethylases, and many RNA-binding proteins (Tong et al., 2018; Xiao et al., 2016; Zaccara et al., 2019). An increasing number of studies have implicated m^6^A modification in a wide range of processes, including early development (Roundtree et al., 2017), immunological response (Shulman and Stern-Ginossar, 2020; Tong et al., 2018), cell differentiation, homeostasis, and response to stress (Shulman and Stern-Ginossar, 2020). Moreover, its dysregulation is associated with a variety of disorders and diseases, such as type 1 diabetes (Wang et al., 2022), esophageal squamous cell carcinoma (Zou et al., 2021), and rheumatoid arthritis (Wu et al., 2021). These findings indicate that m^6^A modification plays an important role in the pathogenesis and development of inflammatory and proliferative diseases. However, the role of m^6^A modification in the pathogenesis of middle ear cholesteatoma remains unclear, and studies directly addressing these factors in middle ear cholesteatoma are lacking. Therefore, this study aimed to investigate the patterns of m^6^A modification in middle ear cholesteatoma.

## 2 Materials and methods

### 2.1 Patients and samples

Five samples of acquired middle ear cholesteatoma tissues were obtained from patients, who underwent cholesteatoma surgery between January 2021 and December 2021, for m^6^A mRNA epitranscriptomic microarray analysis. Meanwhile, five normal post-auricular skin samples were collected and used as controls. Moreover, another 10 acquired middle ear cholesteatoma samples and 10 normal post-auricular skin samples were collected to validate the m^6^A modification level. All samples were immediately stored in liquid nitrogen. This study was approved by the Ethics Committee of The Second Xiangya Hospital, and written informed consent was obtained from all participants.

### 2.2 Total RNA extraction

Total RNA was extracted using TRIzol reagent (Invitrogen, USA), according to the manufacturer’s instructions. Next, the RNeasy Mini Kit (QIAGEN, Germany) was used to purify the RNAs. Total RNA from each sample was quantified using the NanoDrop ND-1000 (Thermo, USA); the OD A260/A280 ratio should be close to 2.0 for pure RNAs (ratios between 1.8 and 2.1 are acceptable), and the OD A260/A230 ratio should be more than 1.8. The RNA integrity was assessed by denaturing agarose gel electrophoresis, and the 28S and 18S ribosomal RNA bands should be fairly sharp, intense bands. The intensity of the upper band should be approximately twice the lower band intensity.

### 2.3 m^6^A immunoprecipitation

Total RNA (3–5 ug) and an m^6^A spike-in control mixture were immunoprecipitated using 2 µg anti-m^6^A rabbit polyclonal antibody (Synaptic Systems, Gottingen, Germany, Cat. No. 202003), and the reaction was incubated with head-over-tail rotation at 4°C for 2 h. Then, 20 μL of Dynabeads™ M-280 Sheep Anti-Rabbit IgG suspension (Invitrogen, Carlsbad, CA, USA, Cat. No. 11203D) per sample was blocked with freshly prepared 0.5% bovine serum albumin (BSA) at 4°C for 2 h. The enriched RNA was eluted with 200 μL of elution buffer (10 mM Tris-HCl, pH 7.4, 1 mM EDTA, 0.05% SDS, 40U proteinase K, and 1 μL RNase inhibitor) at 50°C for 1 h. RNA was extracted using acid phenol–chloroform and ethanol precipitations. qPCR of the positive and negative m^6^A spike-in controls was carried out to check the MeRIP enrichment efficiency. CTA850 (positive control) was used to determine the exogenous RNA, where m^6^A modification occurred, and CTA650 (negative control) was used to identify the exogenous RNA in the absence of m^6^A modification.

### 2.4 Labeling and microarray hybridization

The immunoprecipitated (IP) fraction containing m^6^A-modified RNAs was eluted from the IP magnetic beads, whereas the supernatant (Sup) fraction containing the m^6^A-unmodified RNA was recovered from the centrifuged Sup. Then, IP and Sup RNAs were labeled with Cy5 and Cy3, respectively, using the Arraystar Super RNA Labeling Kit (Arraystar, Rockville, MD, USA, Cat. No. AL-SE-005). These cRNAs labeled with a fluorescent dye were merged and hybridized in Human Arraystar mRNA and lncRNA Epitranscriptomic Arrays (8 × 60 K, Arraystar) that contained 35,175 mRNAs. The hybridized arrays were washed, fixed, and scanned using the Agilent Scanner G2505C (Agilent Technologies, Santa Clara, CA, USA).

In the experiment, 1 μg RNA was used for labeling. The specific activity (pmol dyes per μg cRNAs) of the labeled RNA was obtained using the following formula:

$$Specific\;Activity=\frac{\left(pmol\;per\;\mu l\;dye\right)}{\left(\mu g\;per\;\mu l\;cRNA\right)}.$$

For two colors, if the yield was <825 ng and the specific activity was <8.0 pmol Cy3 or Cy5 per μg cRNA, the hybridization step was not carried out. For one color, if the yield was <1.65 μg and the specific activity was <9.0 pmol Cy3 or Cy5 per μg cRNA, the hybridization step was not carried out.

### 2.5 m^6^A mRNA epitranscriptomic microarray analysis

Agilent Feature Extraction software (version 11.0.1.1) was used to analyze the acquired array images. Raw intensities of IP (Cy5-labeled) and Sup (Cy3-labeled) were normalized to the average of log_2_-scaled spike-in RNA intensities. The “m^6^A quantity” was calculated for the m^6^A modification amount based on IP (Cy5-labeled) normalized intensities. The m^6^A quantity was calculated for the m^6^A methylation amount of each transcript based on the IP (Cy5-labeled) normalized intensities.

$$m6A\;quantity={IP}_{Cy5\;normalized\;intensity.}$$

The m^6^A quantity was calculated based on the normalized intensities of IP signals (the raw signals of Cy5-labeled IP RNA were normalized with the average log_2_-scaled spike-in RNA intensities).

$${IP}_{Cy5\;normalized\;intensity}=\log2\left({IP}_{Cy5\;raw}\right)-Average\left[\log2\left({IP}_{spike-in_{Cy5}\;raw}\right)\right].$$

Differentially m^6^A-modified mRNAs between Cy5-labeled IP and Cy3-labeled Sup groups were identified using the fold change (FC) and statistical significance (*p*-value) thresholds. The percentage of transcripts with m^6^A modifications was calculated based on the normalized intensities of both Cy5-labeled IP and Cy3-labeled Sup samples. The expression levels of mRNAs were calculated based on the total normalized intensities of Cy5-labeled and Cy3-labeled RNAs.

### 2.6 Methylated RNA immunoprecipitation coupled with quantitative real-time PCR

MeRIP–qPCR was performed to further validate the m^6^A modification levels of differentially methylated mRNAs in 10 middle ear cholesteatoma tissue and 10 normal skin samples. First, total RNA was fragmented, and the fragmented RNA was divided into two parts. Then, the large portion of fragmented RNA was immunoprecipitated with the anti-m^6^A antibody, while the small portion was saved as input RNA without immunoprecipitation. The RT-qPCR analysis of IP sample RNAs and the input RNA was subsequently performed. The PrimeScript RT reagent kit (TaKaRa, Tokyo, Japan, Cat. No. RR037A) was used to generate cDNA, and the Arraystar SYBR^®^ Green qPCR Master Mix (Arraystar, Rockville, MD, USA, Cat. No. ASMR-006-5) was used for quantification. Three replicates were tested for each sample. Data were normalized to the input RNA. CTA850 (positive control) and CTA650 (negative control) were used for qPCR normalization. The specific amplification primer pairs are listed in Table 1.

**TABLE 1:** Primers for RT-qPCR validation.

Gene name	Primer sequence
*CD27*	F:5′ GGC​ACT​GTA​ACT​CTG​GTC​TTC 3′
	R:5′ ACT​GAC​ATA​AGG​TAA​GTG​GGT​G 3′
*CSMD1*	F:5′ GAA​ACT​CGA​TGT​CTG​GCT​GG 3′
	R:5′ CAC​TGC​CAT​TAG​TGA​ATC​CG 3′
*USP44*	F:5′ GGT​CAG​GAC​GTA​ATA​ACC​GAG​AG 3′
	R:5′ GCG​GAC​AAG​TCA​TAG​ATA​AAG​CAT 3′
*TIAL1*	F:5′ CCT​AAT​CAT​CTT​ATT​CAG​CCT​ATC​C 3′
	R:5′ GCC​TTC​CTT​TCG​CCA​CTC​T 3′
*MUC12*	F:5′ CAG​CAT​ACA​AGC​AAT​GAC​CCA 3′
	R:5′ GGA​CTC​AAA​TCC​CCA​ACA​AAC 3′

F, forward; R, reverse.

### 2.7 Gene Ontology and Kyoto Encyclopedia of Genes and Genomes pathway analyses

To preliminarily investigate the potential biological function and molecular pathways of differentially m^6^A-modified mRNAs in middle ear cholesteatoma, GO classification and KEGG pathway analyses were performed on the presumptive target genes of differentially m^6^A-modified mRNAs. GO analysis elucidates the functions of differentially m^6^A-modified mRNAs by associating them with certain enriched gene ontological functions and GO terms (http://www.geneontology.org), while KEGG pathway analysis identifies the possible molecular pathways of differentially m^6^A-modified mRNAs involved in cholesteatoma by associating them with certain enriched biological pathways. The statistical significance is calculated by Fisher’s exact test based on the *p*-value and enrichment score (−log_10(p)_). A *p*-value <0.05 indicates that the function or pathway is significant for the physiological or pathological process.

### 2.8 Statistical analysis

Differentially m^6^A-modified mRNAs between two comparison groups were identified by filtering them using the FC and statistical significance (*p*-value) thresholds (FC ≥3 or ≤1/3, *p* <0.05). Hierarchical clustering was performed using R software. GO analysis was performed using the topGO package in the R environment for statistical computing and graphics, and pathway analysis was carried out using Fisher’s exact test. The statistical significance of the enrichment was calculated by Fisher’s exact test based on the *p*-value and −log_10(p)_ transformed as the enrichment score. A *p*-value <0.05 was considered to be statistically significant.

## 3 Results

### 3.1 Differentially methylated or expressed transcripts in cholesteatoma

**FIGURE 1:**
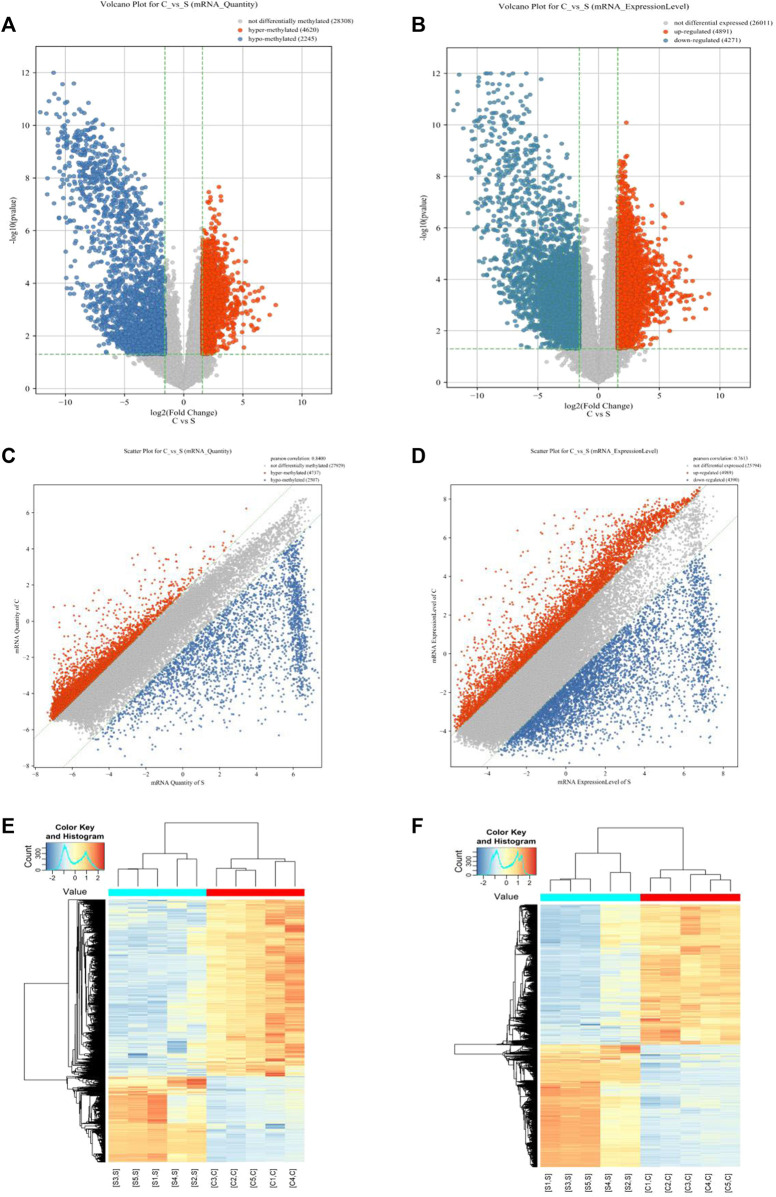
Overview of the m^6^A methylation landscape and mRNA expression profile. **(A)** Volcano plot showing differentially m^6^A-modified mRNAs. **(B)** Volcano plot showing differentially expressed mRNAs. **(C)** Scatter plot showing differentially m^6^A-modified mRNAs. **(D)** Scatter plot showing differentially expressed mRNAs. **(E)** Hierarchical clustering revealing a distinct mRNA methylation pattern between the middle ear cholesteatoma and normal skin samples. **(F)** Hierarchical clustering revealing a distinct mRNA expression pattern between the middle ear cholesteatoma and normal skin samples.

In this study, 35,175 mRNAs were detected using microarray analysis. Compared with the normal skin group, 6,865 mRNAs were significantly differentially methylated in the cholesteatoma group, including 4,620 hypermethylated (FC ≥3, *p* <0.05) and 2,245 hypomethylated mRNAs (FC ≤1/3, *p* <0.05) (Figures 1A, C). In addition, we identified 9,162 mRNAs differentially expressed in the cholesteatoma group compared to the normal skin group, including 4,891 upregulated mRNAs (FC ≥3, *p* <0.05) and 4,271 downregulated mRNAs (FC ≤1/3, *p* <0.05) (Figures 1B, D). Hierarchical clustering identified the interrelationships between the samples and showed distinct m^6^A modification patterns as well as expression patterns of mRNAs between cholesteatoma and normal skin groups (Figures 1E, F).

### 3.2 Association between m^6^A modification and mRNA expression

**FIGURE 2:**
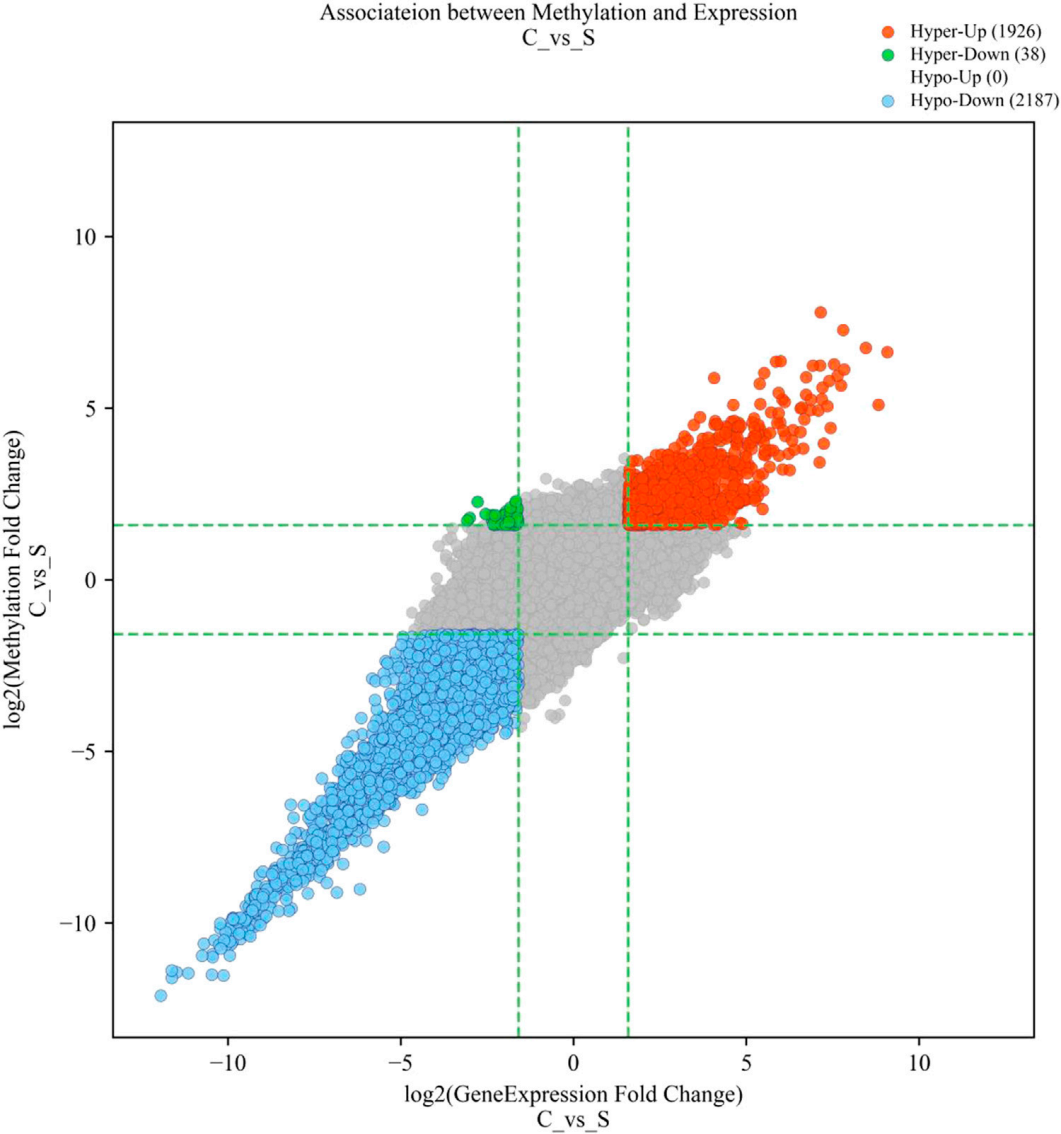
Four-quadrant diagram of mRNAs with significant differences in both m^6^A methylation and mRNA expression. The red dots represent hypermethylated–upregulated mRNAs, the green dots represent hypermethylated–downregulated mRNAs, and the blue dots represent hypomethylated–downregulated mRNAs.

We identified three modes of intersection of differentially expressed mRNAs and differentially m^6^A-modified mRNAs. The expression of 1,926 hypermethylated mRNAs was upregulated, whilst the expression of 2,187 hypomethylated mRNAs was downregulated, and 38 hypermethylated transcripts were downregulated (Figure 2). However, no intersection between hypomethylation and upregulation was observed (Figure 2).

### 3.3 Potential functions and pathways of differentially methylated mRNAs

**FIGURE 3:**
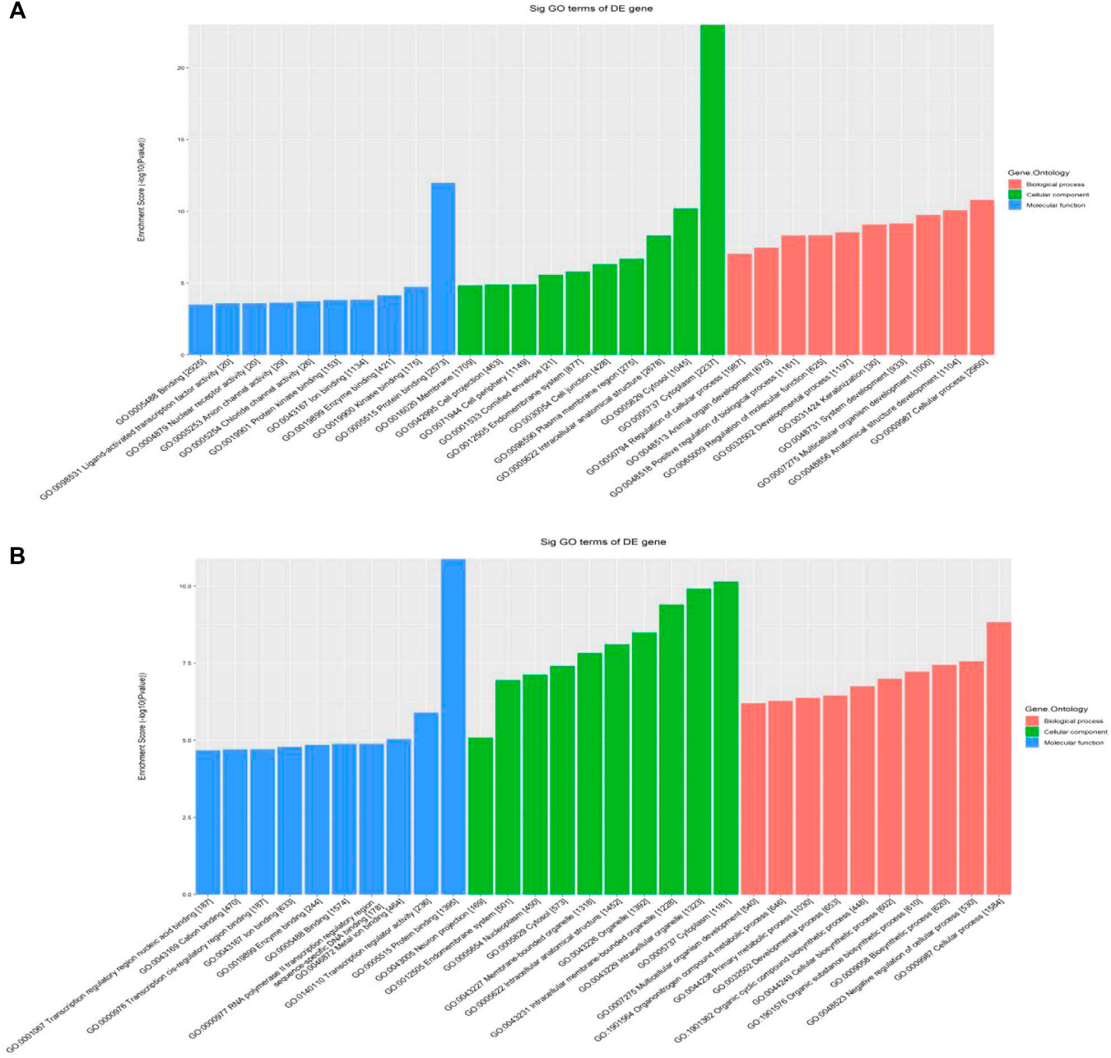
GO classification of differentially methylated mRNAs. **(A)** Top 10 enriched GO items of differentially hypermethylated mRNAs. **(B)** Top 10 enriched GO items of differentially hypomethylated mRNAs.

**FIGURE 4:**
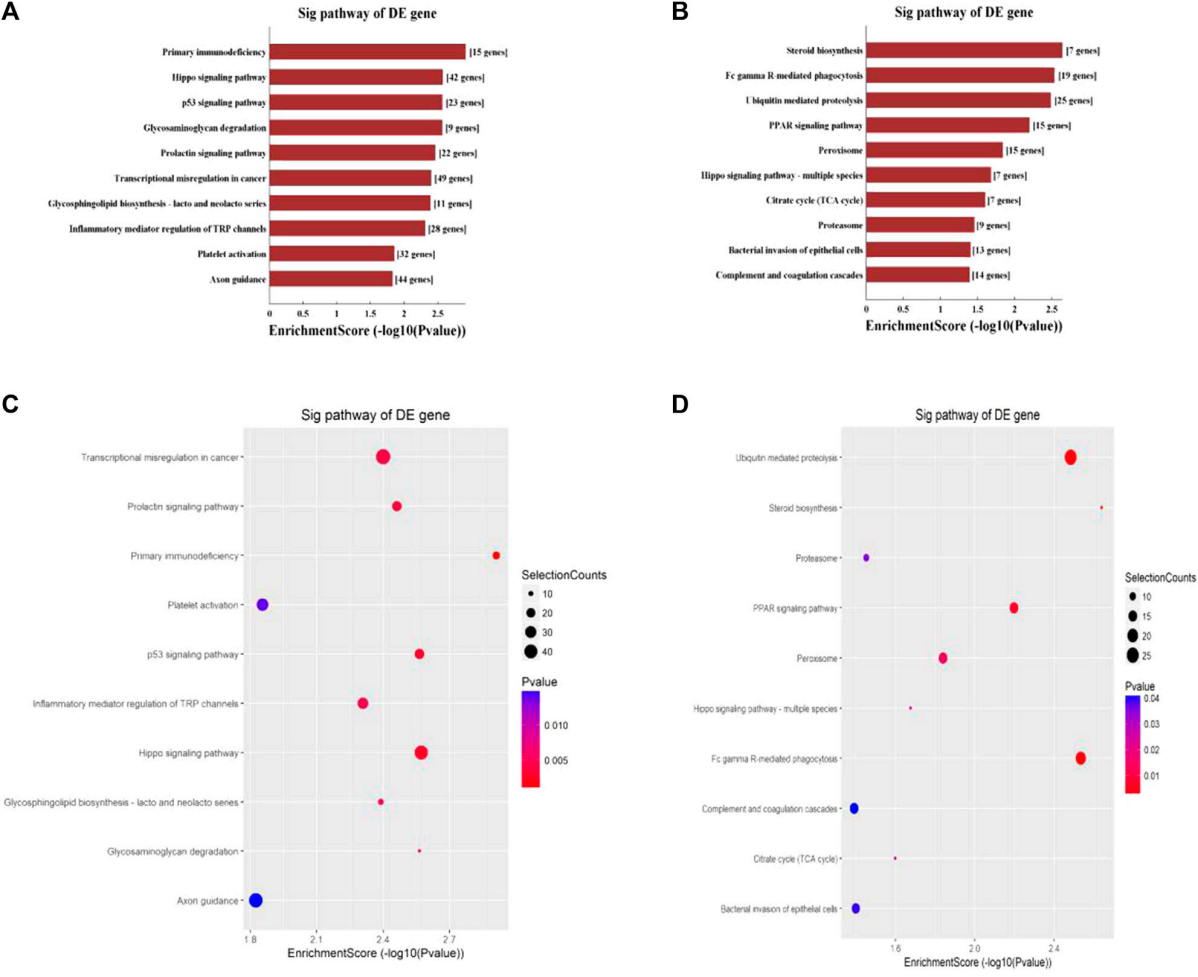
KEGG pathway analysis of differentially methylated mRNAs. **(A)** Top 10 enriched pathways of differentially hypermethylated mRNAs. **(B)** Top 10 enriched pathways of differentially hypomethylated mRNAs. **(C)** Top 10 enriched pathways of differentially hypermethylated mRNAs. **(D)** Top 10 enriched pathways of differentially hypomethylated mRNAs.

Hypermethylated mRNAs were enriched in 1,337 GO terms and 26 KEGG pathways. GO analysis showed that hypermethylated mRNAs were widely distributed in the cytoplasm, cytosol, and endomembrane system and were involved in cellular processes, keratinization, regulation of molecular function, and cell differentiation by binding to proteins, kinases, and enzymes (Figure 3A). KEGG pathway enrichment analysis showed that hypermethylated mRNAs were primarily associated with primary immunodeficiency, the Hippo signaling pathway, the p53 signaling pathway, transcriptional dysregulation in cancer, and inflammatory mediator regulation of transient receptor potential (TRP) channels, among others (Figures 4A, C).

Hypomethylated mRNAs were enriched in 1,079 GO terms and 13 KEGG pathways. GO analysis revealed that hypomethylated mRNAs were mainly distributed in the cytoplasm, intracellular organelles, and intracellular membrane-bound organelles. These mRNAs participate in the regulation of cellular processes, biosynthetic processes, developmental processes, and primary metabolic processes by binding to multiple signaling molecules and regulators (Figure 3B). KEGG analysis showed that hypomethylated mRNAs were mainly related to 13 pathways, including bacterial invasion of epithelial cells, steroid biosynthesis, Fc gamma R-mediated phagocytosis, ubiquitin-mediated proteolysis, and the PPAR signaling pathway (Figures 4B, D).

### 3.4 Validation of differentially m^6^A-modified mRNAs using MeRIP–qPCR

**FIGURE 5:**
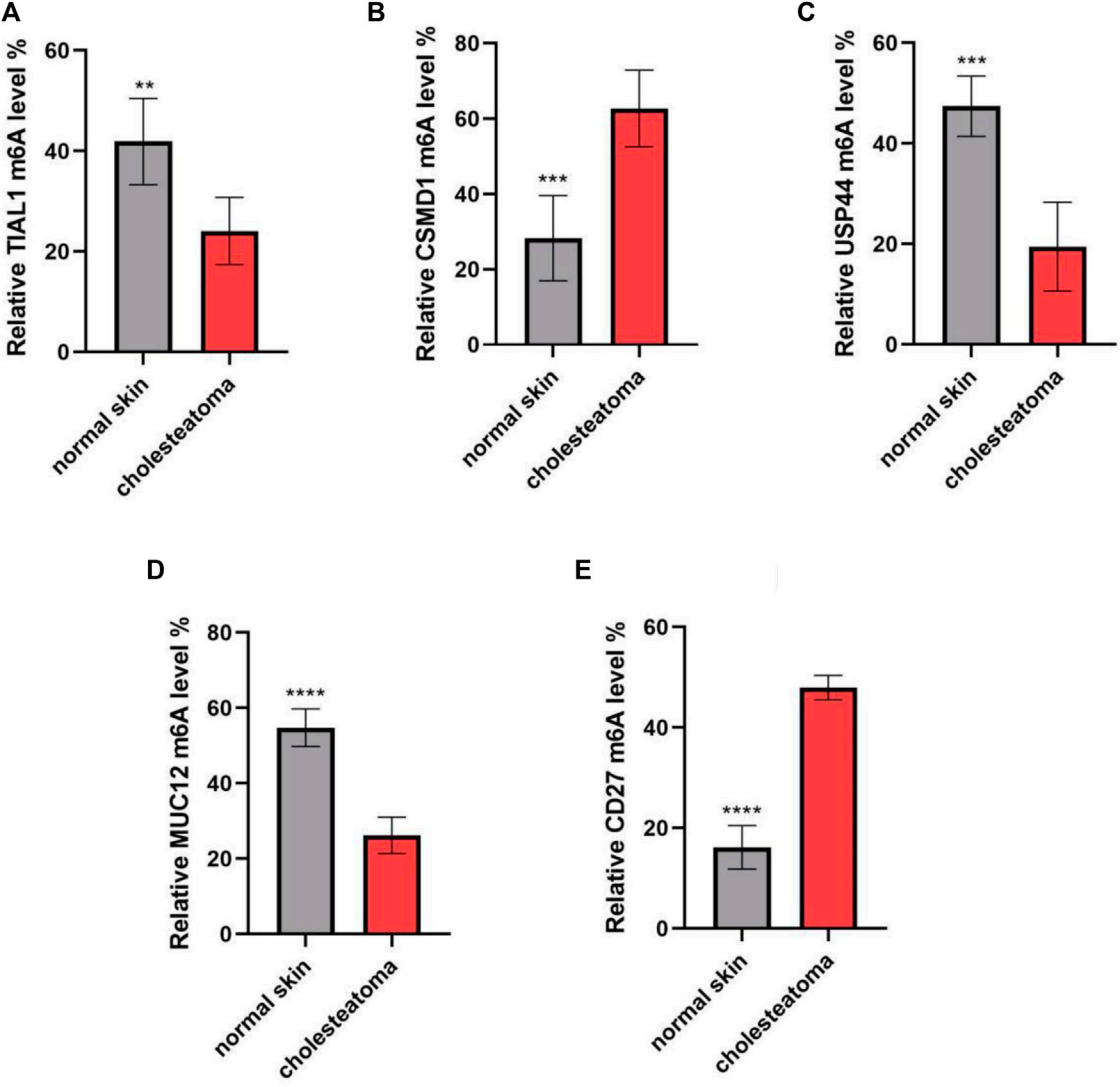
m^6^A methylation levels of key immunity-related genes verified by MeRIP–qPCR. **(A)** *TIAL1*, **(B)** *CSMD1*, **(C)** *USP44*, **(D)** *MUC12*, and **(E)** *CD27*. *n* = 10 per group; **p <0.01, ***p <0.001, and ****p <0.0001.

To validate the microarray results, five differentially m^6^A-modified mRNAs (ENST00000537824, ENST00000266557, ENST00000258499, ENST00000436547, and ENST00000536621) were selected for MeRIP–qPCR (Table 2). The results were consistent with the microarray data: two mRNAs (ENST00000537824 and ENST00000266557) were significantly hypermethylated in middle ear cholesteatoma (*p* <0.05), while the m^6^A modifications of ENST00000258499, ENST00000436547, and ENST00000536621 were significantly decreased (*p* <0.05) (Figure 5).

**TABLE 2:** m^6^A methylation levels of proliferation-related or inflammation-related genes in the microarray analysis.

Transcript ID	Gene symbol	Fold change	Regulation	*p*-value
ENST00000266557	*CD27*	3.270	Hyper	0.0009
ENST00000537824	*CSMD1*	4.559	Hyper	0.0020
ENST00000258499	*USP44*	0.001	Hypo	0.0000
ENST00000436547	*TIAL1*	0.023	Hypo	0.0004
ENST00000536621	*MUC12*	0.002	Hypo	0.0000

## 4 Discussion

Middle ear cholesteatoma is a pathological condition associated with active proliferation of epithelial cells under inflammation, which can result in various complications by gradually expanding to the middle ear and temporal bone (Kuo, 2015; Yamamoto-Fukuda et al., 2018). m^6^A RNA modification is the most predominant and well-studied mRNA modification that regulates gene expression at the post-transcriptional level, participating in a variety of physiological and pathological processes (Tong et al., 2018; Xiao et al., 2016; Zaccara et al., 2019). However, the potential role of m^6^A in cholesteatoma development has not been studied extensively. Therefore, in this study, we analyzed m^6^A modification patterns in middle ear cholesteatoma.

Microarray analysis data demonstrated a distinct m^6^A modification pattern in middle ear cholesteatoma and normal skin samples. A total of 4,620 hypermethylated mRNAs and 2,245 hypomethylated mRNAs were identified in cholesteatoma compared with normal skin samples. Mounting evidence suggests that aberrant m^6^A modification levels contribute to diverse diseases, such as diabetes, esophageal squamous cell carcinoma, and rheumatoid arthritis (Wang et al., 2022; Zou et al., 2021; Wu et al., 2021). The differential m^6^A modification pattern in our study indicated that m^6^A modification of mRNAs may play a significant role in the pathogenesis and development of cholesteatoma.

Potential biological functions and signaling pathways enriched with differentially m^6^A-modified mRNAs were explored in this study. GO analysis showed that differentially methylated mRNAs were associated with cellular processes, cell differentiation, biosynthetic processes, cell cycle, regulation of molecular function, and keratinization. Accumulating evidence has demonstrated that m^6^A modification is crucial for the development, differentiation, activation, and homeostasis of cells, indicating that the dysregulation of m^6^A can result in the pathogenesis and development of some diseases (Ge et al., 2022). KEGG pathway enrichment analysis showed that hypermethylated mRNAs were primarily associated with the Hippo signaling pathway, the p53 signaling pathway, and inflammatory mediator regulation of TRP channels, whereas hypomethylated modified mRNAs were mainly related to bacterial invasion of epithelial cells and the Hippo signaling pathway.

Notably, we found that both hypermethylated and hypomethylated mRNAs were enriched in some biological processes and signaling pathways. For instance, hypermethylated mRNAs were enriched in inflammatory mediator regulation of TRP channels, whereas hypomethylated modified mRNAs were enriched in bacterial invasion of epithelial cells, indicating that hypermethylated mRNAs and hypomethylated mRNAs were jointly involved in the inflammatory response of cholesteatoma. Indeed, recent studies have shown that TRP channels are associated with infections (Pinho-Ribeiro et al., 2018; Scheraga et al., 2020). Moreover, we found that the Hippo signaling pathway was present in the enrichment results for both hypermethylated and hypomethylated mRNAs. This signaling pathway is involved in cell growth, differentiation, proliferation, and apoptosis and plays a key role in organ size control, tissue regeneration, and tumor development (Ahmad et al., 2022). The yes-associated protein (YAP) acts as a downstream negative regulator of the Hippo pathway, controlling cell proliferation, differentiation, and cancer metastasis (Cunningham and Hansen, 2022). Interestingly, studies have found that the expression and nuclear translocation of YAP increased in middle ear cholesteatoma, which was involved in the proliferation and formation of cholesteatoma (Akiyama et al., 2017; Yamamoto-Fukuda et al., 2020). Therefore, it is necessary to elucidate the role of the Hippo pathway in the pathophysiology of middle ear cholesteatoma. The p53 signaling pathway is also closely related to cell proliferation and apoptosis. For example, APOBEC3B promotes the proliferation and migration of cervical cancer cells and inhibits their apoptosis via the p53 pathway (Wei et al., 2022). In addition, previous studies showed that the expression of p53 protein increased in the cholesteatoma epithelium and may contribute to the reaction via cellular hyperproliferation (Fukuda et al., 2021). Based on these findings, m^6^A may participate in cholesteatoma pathogenesis through proliferation-related and inflammation-related pathways.

Excessive proliferation of squamous epithelial cells and local inflammatory response are the two major clinical features of middle ear cholesteatoma, which are closely related to its occurrence and development. Therefore, in our study, we selected certain proliferation-related or inflammation-related genes from a large number of genes with differential m^6^A modification differences for further analysis. Although the genes selected in this study have not currently been explored in middle ear cholesteatoma, they could provide directions and targets for the subsequent search for the non-pharmaceutical treatment of middle ear cholesteatoma. Ubiquitin-specific peptidase 44 (USP44) is a member of a family of deubiquitinating enzymes that play an important role in cell proliferation (Lou et al., 2022). In this study, we found that USP44 was hypomethylated and downregulated in cholesteatoma compared to that in the control, indicating that USP44 may play an essential role in cholesteatoma development. Zhang et al. showed that the expression level of USP44 was markedly decreased in colorectal cancer, and USP44 overexpression inhibited proliferation and enhanced apoptosis in colorectal cancer cells (Huang et al., 2020). A recent study demonstrated that USP44 overexpression inhibited the proliferation and migration of clear cell renal cell carcinoma cell lines; inversely, USP44 knockdown exerted opposite effects (Zhou et al., 2020). As a deubiquitinating enzyme, cylindromatosis (CYLD) was significantly downregulated in cholesteatoma and may be involved in cholesteatoma epithelial hyperplasia (Byun et al., 2010). Moreover, a study revealed that the level of CYLD expression in acquired cholesteatoma is significantly correlated with the clinicopathological characteristics, including wound healing, infection, and recurrence in cholesteatoma patients (Miyake et al., 2020). These studies suggest that deubiquitinating enzymes may be involved in the formation of cholestomas. However, currently, there are no relevant studies assessing the role of USP44 in the development of cholesteatoma. Therefore, we hypothesized that the hypermethylation of USP44 results in its downregulation in cholesteatoma, which may participate in the hyperproliferation of keratinocytes in cholesteatoma. This presupposition and the specific mechanism will be investigated in future studies.

Moreover, T-cell intracellular antigen 1-related/like protein (TIAR/TIAL1) and MUC12 were significantly hypomethylated in our microarray results, accompanied by downregulation. TIAL1 is a DNA/RNA-binding protein involved in the transcriptional and post-transcriptional regulation of gene expression and plays a vital role in human physiology and pathology (Sánchez-Jiménez and Izquierdo, 2015; Izquierdo et al., 2011). A previous study showed that knockdown of TIAL1 results in increased cell proliferation, tumor growth, and invasion, Moreover, the TIAL1 protein was shown to be downregulated in many tumors of epithelial origin, including skin, breast, and colon (Izquierdo et al., 2011). As for MUC12, it is a member of the mucin family and participates in cancer progression, owing to its capacity to transduce intracellular signaling (Gao et al., 2020; Matsuyama et al., 2010). Gao et al. (2020) showed that overexpression of MUC12 increased cell growth and invasion, whereas deficiency of MUC12 exerted opposite effects on renal cell carcinoma cells. In contrast, MUC12 was significantly downregulated in colorectal cancer tissues, and low MUC12 expression was accompanied by worse disease-free survival in patients with stage II or III colorectal cancer (Matsuyama et al., 2010). It has been confirmed that the abundant mucin in the perimatrix of cholestomas, associated with its inflammatory response, may play a potential role in the proliferation of keratinized epithelial cells and bone destruction (Nagai et al., 1992; Nagai et al., 1993; Nagai et al., 2006). However, whether TIAL1 and MUC12 are involved in the pathogenesis of cholesteatoma requires further investigation.

However, our study has some limitations. First, the number of validated clinical specimens was small. Second, all candidates have not been explored at the cellular level and with animal models, and their specific role and mechanism in middle ear cholesteatoma have not been explored. In the future, we will validate a large number of clinical samples. Moreover, further mechanistic studies on cell lines and animal models would be conducted to investigate the exact mechanism of these proliferation-related or inflammation-related genes in the pathogenesis of middle ear cholesteatoma.

In conclusion, our study first identified m^6^A modification patterns in middle ear cholesteatoma and explored the potential biological functions and pathways associated with its pathogenesis. Our study suggests that m^6^A modification may serve as a biological marker and a putative target for the diagnosis and treatment of cholesteatoma.

## Data Availability

The original contributions presented in the study are included in the article/Supplementary Materials; further inquiries can be directed to the corresponding author.
